# Changes of cardiac output and velocity time integral in blood return at the end of renal replacement therapy predict fluid responsiveness in critically Ill patients with acute circulatory failure

**DOI:** 10.1186/s12871-023-01976-7

**Published:** 2023-01-14

**Authors:** Daozheng Huang, Jie Ma, Shouhong Wang, Tiehe Qin, Feier Song, Tieying Hou, Huan Ma

**Affiliations:** 1 Department of Critical Care Medicine, Guangdong Provincial Geriatrics Institute, Guangdong Provincial People’s Hospital (Guangdong Academy of Medical Sciences), Southern Medical University, Guangzhou, 510080 China; 2 Medical Department, Guangdong Provincial People’s Hospital (Guangdong Academy of Medical Sciences), Southern Medical University, Guangzhou, 510080 China; 3 Department of Critical Care Medicine, Jiangmen Central Hospital, Jiangmen, 529000 China; 4 The Second School of Clinical Medicine, Southern Medical University, Guangzhou, 510080 China; 5 Department of Emergency Medicine, Guangdong Provincial People’s Hospital (Guangdong Academy of Medical Sciences), Southern Medical University, Guangzhou, 510080 China; 6 Guangdong Clinical Laboratory Center, Guangdong Provincial People’s Hospital (Guangdong Academy of Medical Sciences), Southern Medical University, Guangzhou, 510080 China

**Keywords:** Fluid responsiveness, Blood return, Blood infusion test, Passive leg raise, Velocity time integral, Cardiac output

## Abstract

**Objectives:**

To observe if blood return, also defined as the blood infusion test (BIT) could predict fluid responsiveness in critically ill patients with acute circulatory failure and renal replacement therapy (RRT).

**Methods:**

This was a single-center, prospective, diagnostic accuracy study. Before BIT, the passive leg raise test (PLRT) was performed to record the change of cardiac output (ΔCO) by pulse contour analysis, and ΔCO >  = 10% was defined as the fluid responder. Meanwhile, the change in velocity time integral (ΔVTI) was recorded by ultrasound. Later, the ΔCO and ΔVTI during BIT were recorded 5–10 min after PLRT. The receiver-operating characteristic curves of ΔCO and ΔVTI of BIT were performed in predicting the fluid responder defined by PLRT.

**Results:**

A total of 43 patients with acute circulatory failure undergoing RRT were enrolled in the present study, and 25 patients (58.1%) were recognized as responders during PLRT. According to the receiver-operating characteristic curves, the cutoff value of ΔCO was 10% and ΔVTI was 9% during BIT with the area under curve of 0.96 and 0.94, respectively.

**Conclusions:**

BIT in RRT could identify fluid responsiveness in critically ill patients with shock.

**Trial registration:**

ChiCTR-DDD-17010534. Registered on 30/01/2017 (retrospective registration).

**Supplementary Information:**

The online version contains supplementary material available at 10.1186/s12871-023-01976-7.

## Background

Patients with renal injury and shock happened almost every day in the intensive care unit (ICU). According to Hoste et al., hypotension happened in 47.6 per 100 ICU adult patients who had a high risk of acute kidney injury with 53.8% [1]. Shock/hypotension is attributed to kinds of reasons, one of which is hypovolemia. Dynamic tests, like the passive leg raise test (PLRT) and infusion of small volumes of fluid [2], have been widely used to observe the changes in cardiac output (CO) to identify fluid responsiveness. These tests could induce short-term changes in cardiac preload which are dependent on the heart–lung interaction. Kinds of techniques could obtain the hemodynamic evaluations during the tests above, including echocardiography [3] and thermodilution [4] which are used broadly in intensive care units.

Although renal replacement therapy (RRT) has potent effects on fluid management, they cannot have the patient with the ‘optimal volume’ during the course of treatment every time. When it has to be terminated due to kinds of reasons, the patient is probable of being from insufficient volume or overload. The future direction of fluid management would have a setting specific aim if we could tell the insufficient volume at the end of RRT. We have already found that the blood pump-out test at the initial procedure of RRT could serve as a complementary maneuver to predict fluid responsiveness [5]. Blood return has a similar effect on fluid expansion due to about 200 ml of blood going back to the body. We hypothesized that the procedure of venous blood return, also known as the blood infusion test (BIT) could also be another supplemental method to predict fluid responsiveness. The present study aimed to evaluate if the change of CO and velocity time integrals (VTI) during BIT could identify fluid responders from critically ill patients with shock.

## Methods

This was a single-center, prospective, diagnostic accuracy study in an intensive care unit (ICU) of Guangdong Provincial People’s Hospital (Registration No. ChiCTR-DDD-17010534). It was approved by the Ethical Committee (No. GDREC2016313H) and informed consent was obtained from all included participants or their immediate family members.

### Patients

Patients who met the following criteria were included: 1) ≥ 18 years old, 2) acute circulatory failure, 3) undergoing RRT, 4) transpulmonary thermodilution device (Pulse Contour Cardiac Output 2 (PiCCO2) device, Pulsion Medical Systems, Munich, Germany) already in place. Patients with pregnancy and end-stage malignant tumors were excluded.

Acute circulatory failure was defined as 1) systolic arterial pressure < 90 mmHg or the mean arterial pressure < 70 mmHg, with associated tachycardia, or 2) use of vasopressors. Clinical signs of tissue hypoperfusion and hyperlactatemia might be typically present, including urine < 0.5 ml/kg/h for more than two hours, heart rate > 100 beats per minute, skin tinea, and lactate > 1.5 mmol/L [6].

All patients were treated with mechanical ventilation with spontaneous breathing. All patients were treated with continuous venovenous hemodiafiltration (CVVHDF) or continuous venovenous hemofiltration (CVVH). All patients received intensive care. After resuscitation, the patients were at the stage of stabilization and de-escalation [7]. At this stage, the goal lay in organ support and individualized fluid management. International guidelines recommended that following initial fluid resuscitation, additional fluids be guided by frequent reassessment of hemodynamic status [8].

### Study protocol

**Fig. 1:**
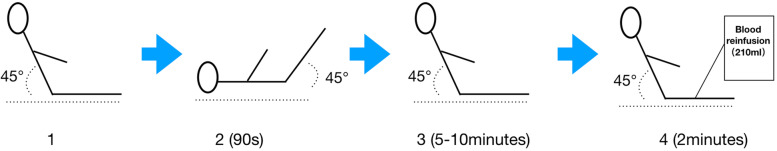
Graphic description of the study protocol

All enrolled patients would go through a two-step procedure, comprising PLRT and BIT. An ICU physician and a sonologist perform the measurement simultaneously. At the end of RRT before blood infusion, hemodynamic measurement was performed via thermodilution (baseline intrathoracic blood volume index, global end-diastolic volume index, systemic vascular resistance index, extravascular lung water index) and pulse contour analysis (baseline CO, cardiac index, stroke volume, stroke volume index, stroke volume variation) in the 45° semi-recumbent position. The sonologist performed the ultrasound examination concurrently. The bed was then lowered and the patient’s legs were elevated to 45°. During 45 s, the second measurement was taken, including the maximal CO and maximal VTI. Later, re-assessments of the CO and VTI were performed (BIT baseline) on patients in the 45° semi-recumbent position. It was usually taken five to ten minutes when parameters were recorded. Amid every position change, hemodynamic parameters were recorded accordingly. The process of PLRT was described in detail in previous literature [9]. BIT, conducted several minutes after PLRT, was the process of blood return from the blood filter and pipelines to the body at the end of RRT (Fig. 1). The BITs were initiated with a flow rate of 100 ml/minute for blood return (lasting about 2 min). The maximal CO and maximal VTI were recorded during blood infusion. The blood pressure transducer of the PiCCO2 device was strapped to the patient's upper arm, keeping it at the same level as the right atrium [10]. All fluids were stopped during both tests and vasopressors were maintained at a constant speed if no dramatic decrease in mean arterial pressure occurred.

Left ventricular outflow tract (LVOT) VTI was performed by the same sonologist using transthoracic echocardiography (Phillips EPIQ5). The optimum signal for velocity measurements in LVOT was acquired from an apical five-chamber view. The blood flow velocity waveforms were collected within 3 consecutive cardiac cycles to calculate an average VTI. Data on the patient’s heart rhythm was collected. The average VTI out of six measurements was used to determine the value in patients with atrial fibrillation.

RRT was performed through a double-lumen catheter inserted into the femoral vein. CVVHDF or CVVH were performed using standard equipment (Fresenius, Germany), with a hemofilter model (Ultraflux AV1000S). Anticoagulation was achieved using a continuous infusion of heparin systemically or citrate regionally. At the end of RRT, blood from the pipelines was reinfused back into the patient with a total volume of 210 ml.

The maximal CO was recorded by PiCCO2 and the maximal LOVT VTI was recorded by echocardiography. The parameters mentioned above were acquired right before and 2 min after PLRTs or BITs. And the change between these two timing was calculated as ΔCO [= (CO _after PLRT/BIT_ –CO _before PLRT/BIT_)/ CO _before PLRT/BIT_] and ΔVTI [= (VTI _after PLRT/BIT_ –VTI _before PLRT/BIT_)/ VTI _before PLRT/BIT_). And the interval between PLRT and BIT was approximately 2 min which was sufficient for COs or VTIs to return to baseline. Patients who had more than or equal to 10% of ΔCO [11] and 15% of ΔVTI [12] were considered as the responder in PLRT. In the present study, we recognized ΔCO ≥ 10% in PLRT as ‘the golden standard’ to predict fluid responsiveness since there were few indications for the actual fluid challenge.

### Statistical analysis

A target sample size of 40 patients was based on a 92.5% sensitivity observed in the pre-experiment and the intention to obtain the statistical significance of α = 0.05, allowing for an error of δ = 0.08. List-wise deletion was performed dealing with missing values. The normality of data was tested by the Kolmogorov–Smirnov normality test and histogram. Continuous variables were expressed as mean ± standard deviation or the interquartile range. The receiver-operating characteristic (ROC) curves were constructed to test the ability of △CO or △VTI during BIT to predict fluid responsiveness. The optimal cutoffs were determined using Youden’s index. R version 4.0.2was used for analysis and 2-sided *P* ≤ 0.05 was considered significant.

## Results

A total of 43 patients were included Baseline characteristics were shown in Table 1, including disease severity, ultrasonic variables, pre-load indices via PiCCO2, laboratory parameters, and use of vasopressors. The median age was 87 years old and 46.5% of the patients were male. The settings and mode of mechanical ventilation were demonstrated. Hyperlactatemia was not predominant and vasopressors were not prescribed in 44.2% of patients. Hemodynamic variables during PLRT and BIT were shown in Table 2. CO increased by 11.8% after PLRT and 8.9% after BIT. Meanwhile, VTI increased by 12.2% after PLRT and 15.3% after BIT. Both CO and VTI ascended after passive leg raise and blood infusion.

**Table 1:** Baseline characteristics

	Total (*N* = 43)	Responders (*n* = 25)	Non-responders (*n* = 18)
Demographics			
Male (%)	20, 46.5%	12, 48.0%	8, 44.4%
Age (years old)	87(67–88)	87(63–89)	88(67–88)
BSA (m^2^)	1.7(1.7–1.8)	1.7(1.7–1.8)	1.7(1.7–1.7)
BMI (kg/m^2^)	22.5(22.0–25.4)	24.2(22.5–25.4)	22.0(20.2–23.3)
Disease severity			
APACHE II scores	24(21–32)	24(21–31)	26(21.25–32)
SOFA scores	13.7 ± 4.2	13.0 ± 4.2	14.6 ± 4.1
Hemodynamics			
LVEF (%)	55(50–60)	55(50–58)	58(55–60)
LVOTD (cm)	1.7(1.6–2.0)	1.8(1.7–2.0)	1.7(1.5–2.0)
Sinus rhythm (%)	32, 74.4%	16, 64.0%	16, 88.9%
Atrial fibrillation (%)	11, 25.6%	9, 36.0%	2, 11.1%
IAP (mmHg)	9.2(7.5–10.8)	8.8(7.0–10.5)	9.7(8.2–11.2)
ITBVI (ml/ m^2^)	1135(921.5–1244.5)	1071(908–1214)	1171(929–1306)
GEDVI (ml/ m^2^)	892.9 ± 183.4	864.4 ± 167.6	932.6 ± 201.5
SVRI (dyn·s/cm^5^/m^2^)	1930(1453–2247)	2017(1587–2841)	1750(1343–2000)
EVLWI (ml/kg)	10.3(8.3–12.1)	10.6(8.6–12.8)	9.55(8.1–11.6)
Laboratory parameters			
Lactic acid (mmol/L)	1.3(1.0–1.6)	1.4(1.2–2.6)	1.2(0.9–1.4)
NT-proBNP (pg/ml)	5615(2704–14,244)	5129(2789–10,642)	6755.5(1582.5–16,051.8)
ScvO_2_ (%)	67.3(55.8–73.3)	67.8(55.4–73.2)	66.7(62.6–74.1)
Blood urea nitrogen (mmol/L)	13.6(8.1–16.2)	15.4(13.5–16.7)	8.4(6.7–11.9)
Serum creatinine (μmol/L)	182.6(104.6–290.3)	217.7(167.0–310.9)	112.1(97.7–182.9)
Mechanical ventilation settings			
Tidal volume(ml/kg)	404(335–477)	409(383–452)	394(261–539)
PEEP (cmH_2_O)	5(5–8)	7(5–8)	5(5–7)
Pressure control (cmH_2_O)	14(8–16)	16(12–16)	8(0–14)
Pressure Support (cmH_2_O)	16.0(12.0–17.5)	14(12–16)	16(8–20)
Mechanical ventilation mode			
SPONT	15, 34.9%	6, 24.0%	9, 50.0%
SIMV	20, 46.5%	15, 60.0%	5, 27.8%
Assist/Control	3, 7.0%	3, 12.0%	0, 0.0%
P-CMV	5, 11.6%	1, 4.0%	4, 22.2%
Vasopressors			
Without Vasopressor	19, 44.2%	9, 36.0%	10, 55.6%
Norepinephrine < 0.2ug/mg/h	3, 7.0%	1, 4.0%	2, 11.1%
Norepinephrine 0.2–0.4ug/mg/h	13, 30.2%	9, 36.0%	4, 22.2%
Norepinephrine > 0.4ug/mg/h	2, 4.7%	2, 8.0%	0, 0.0%
Norepinephrine & Dopamine	2, 4.7%	0, 0.0%	2, 11.1%
Sodium nitroprusside	4, 9.3%	4, 16.0%	0, 0.0%

*BSA* Body Surface Area, *BMI* Body mass index, *APACHE* Acute Physiology and Chronic Health Evaluation, *SOFA* Sequential Organ Failure Assessment, *IAP* Intra-abdominal pressure, *SVRI* Systemic vascular resistance index, *ITBVI* Intrathoracic blood volume index, *GEDVI* Global end-diastolic volume index, *EVLWI* Extravascular lung water index, *ScvO*_*2*_ Central venous oxygen saturation, *NT-proBNP* NT-proB-type Natriuretic Peptide, *PEEP* Positive End-Expiratory Pressure, *SIMV* Synchronized intermittent mandatory ventilation, *P-CMV* Pressure-controlled mandatory ventilation

**Table 2:** Hemodynamic variables during passive leg raise test (PLRT) and blood infusion test (BIT)

	Baseline	After PLRTs	After BITs
SBP (mmHg)	129.5 ± 20.9	139.4 ± 24.2	146.3 ± 23.0
DBP (mmHg)	56.5 ± 8.4	59.9 ± 11.1	60.4 ± 12.2
Heart Rate (beats/minute)	88.4 ± 20.0	90.2 ± 21.9	88.3 ± 20.6
MAP (mmHg)	81.8 ± 11.4	86.0 ± 19.6	91.9 ± 13.0
CVP (cmH_2_O)	9.2 ± 4.9	11.0 ± 4.5	11.2 ± 5.6
PiCCO2			
Cardiac output (CO)(L/min)	5.5 ± 1.9	6.0 ± 2.0	6.0 ± 2.0
△CO (%)	-	11.8(3.5–20.0)	8.9(5.2–17.8)
Cardiac index(L/(min·m^2^))	3.2 ± 1.2	3.5 ± 1.3	3.5 ± 1.3
Stroke volume (ml)	65.8 ± 25.6	70.1 ± 27.0	71.0 ± 26.2
Stroke volume index (ml/m^2^)	39.1 ± 15.7	40.8 ± 16.6	41.8 ± 16.0
Stroke volume variation (%)	8.5(5.0–13.3)	9.0(5.5–11.5)	8.5(5.0–12.0)
Ultrasound Examination			
Maximal IVC (cm)	2.2 ± 0.3	2.2 ± 0.3	2.4 ± 0.2
Minimal IVC (cm)	1.9 ± 0.4	1.9 ± 0.4	2.1 ± 0.3
Velocity time integral (VTI) (cm/s)	33.8(18.7–44.8)	42.6(21.1–47.5)	38.8(23.7–50.3)
△VTI (%)	-	12.2(0.7–26.0)	15.3(2.9–29.9)

*SBP* Systolic blood pressure, *DBP* Diastolic blood pressure, *IVC* Inferior vena cava, *CVP* Central Venous Pressure, *MAP* Mean Artery Pressure; ΔCO [= (CO _after PLRT/BIT_ –CO _before PLRT/BIT_)/ CO _before PLRT/BIT_] and ΔVTI [= (VTI _after PLRT/BIT_ –VTI _before PLRT/BIT_)/ VTI _before PLRT/BIT_)

Using the threshold of ΔCO ≥ 10% and ΔVTI ≥ 15%, a contingency table was drawn to evaluate the accuracy of ΔVTI via echocardiography for determining fluid responsiveness during PLRT, comparing with ΔCO (Table 3). The diagnostic accuracy was measured using sensitivity (72%), specificity (100%), positive predictive value (100%), negative predictive value (72%), negative likelihood ratio (0.28), accuracy (83.7%), and Youden's index (0.72).

**Table 3:** Contingency Table Evaluating the Accuracy of Velocity Time Integral (VTI) via Echocardiography for Determining Fluid Responsiveness during Passive Leg Raise Test (PLRT)

	PiCCO		
Echocardiography	△CO ≥ 10%	△CO < 10%	Total
△VTI ≥ 15%	18	0	18
△VTI < 15%	7	18	25
Total	25	18	43

*PiCCO* Pulse index Contour Cardiac Output, *CO* Cardiac output; ΔCO [= (CO _after PLRT_–CO _before PLRT_)/ CO _before PLRT_] and ΔVTI [= (VTI _after PLRT_ –VTI _before PLRT_)/ VTI _before PLRT_)

**Fig. 2:**
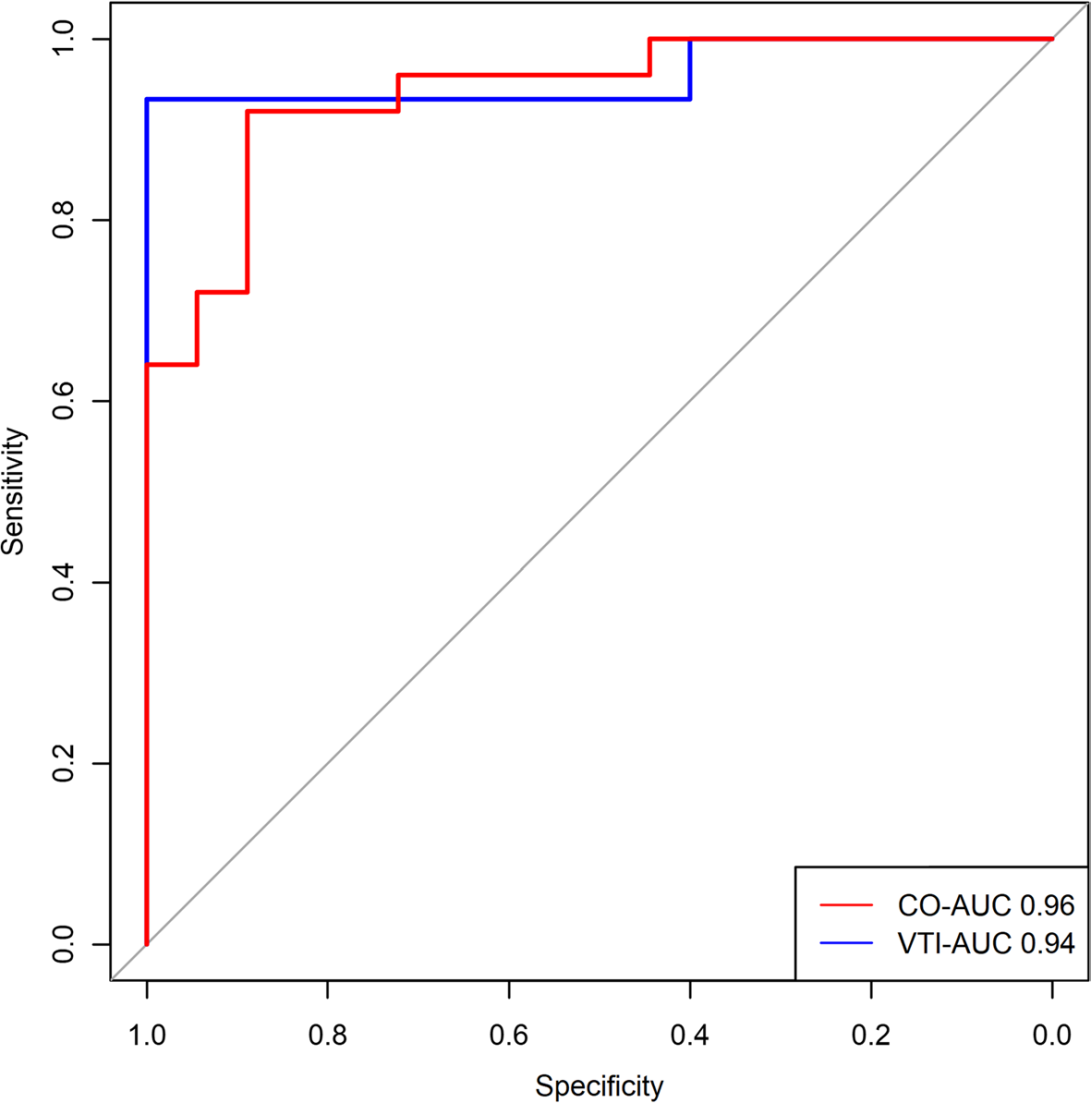
Prediction of fluid responsiveness. The receiver-operating characteristic curves of the changes in cardiac output (ΔCO) (cut-off value 10%, sensitivity 93.3%, specificity 100%) and the changes in velocity time integral (ΔVTI) (cut-off value 9%, sensitivity 92.0%, specificity 83.3%) after blood infusion test

The present study employed ΔCO ≥ 10% during PLRT as the “golden standard” for predicting fluid responsiveness. There were 25 patients (58.1%) identified by ΔCO and 18 patients (41.9%) by ΔVTI to be fluid responders during PLRT. In the ROC analysis, the optimal cutoff value of ΔCO was 10.4% during BIT with a sensitivity of 93.3% and a specificity of 100%. The area under curve was 0.96 (95% CI 0.88–1.00). Meanwhile, the optimal cutoff value of ΔVTI was 8.7% during BIT with a sensitivity of 92.0% and a specificity of 88.9%. The area under curve was 0.94 (95% CI 0.87–1.00) (Fig. 2). Cross tabulation evaluating the accuracy of CO and VTI during BIT for determining fluid responsiveness was shown in Table S1.

## Discussions

According to our study, patients had increased CO and VTI both after PLRT and BIT compared with those before these two tests, which indicated that the change of CO and VTI during BIT could identify fluid responsiveness in circulatory shock patients with RRT.

The evaluation of fluid responsiveness happens all the time in intensive care units which play a vital role in fluid management. Fluid bolus, which could be treated as preload challenge, was classically used to test if it could induce hemodynamic improvement. But it was possible to be overloaded if no attempt was made to evaluate fluid responsiveness with volume expansion. Therefore, static parameters like central venous pressure and dynamic markers such as pulse pressure variation and stroke volume variation were used based on heart–lung interaction [13]. The conventional fluid challenge, giving 1000 ml of crystalloids or 300-500 ml of colloids over 30 min [14, 15], was gradually improved by the mini-fluid challenge (giving 100 ml colloid over 1 min) [16] or low-volume infusion (50 ml crystalloid solution over 10 s) [17]. The current practice of fluid challenge and evaluation of fluid responsiveness in critically ill patients is highly variable [18]. PLRT, taken as a reversible preload challenge, could be repeated frequently without any fluid dripping into the body [19], eliminating the potential risk of additional bolus infusion, and was accurate even in patients spontaneously complicated with cardiac arrhythmias, and low respiratory system compliance [20]. Additionally, an increase in CO after the mini-fluid challenge could also define fluid responsiveness [21]. In our opinion, PLRT was proved to be the most useful and convenient maneuver reported to be reliable consistent with studies, and reversible in preload challenges [2]. Therefore, we adopted the changes of preload during PLRT, expressed by CO and VTI, and △CO as the ‘golden standard’ in our study to test if BIT has the same power to identify the fluid responders from all patients with shock and RRT.

The reliability of CO measurements by pulmonary thermodilution in RRT was challenged because it was found that the thermodilution curve forms were modified resulting in inaccurate calculation of related hemodynamic parameters [22]. But we noticed that the thermal indicator was injected through a dialysis catheter in the abovementioned study which was not a normal way stipulated by factory settings. Additionally, Dr. Dufour and his colleagues confirmed that hemodynamic measurements derived from transpulmonary thermodilution were not affected by RRT [23]. So we could take the change of CO derived from PiCCO2 as the golden standard to recognize fluid responders.

Transthoracic echocardiography was performed excellently in estimating cardiac out based on LVOT-VTI compared with pulmonary artery catheter [24, 25]. Additionally, LVOT-VTI combined with PLRT could screen volume responsiveness from end-stage renal disease patients after hemodialysis with the mean VTI increasing from 30.31 cm to 34.91 cm and the mean ΔVTI between 12.64% and 16.84% [26]. And LVOT-VTI is reliable and repeatable in distinguishing fluid responders from all shock patients. As reported in the study of Lill Bergenzaun and his colleagues [27], LVOT-VTI was the best repeatable echocardiographic parameter in the evaluation of left ventricular systolic function.

Little similar studies were found according to our findings except the one published by our team last year which focused on volume changes during blood pump-out test at the early stage of continued blood purification [5]. Umgelter et al. reported that general hemodynamic parameters, such as heart rate, mean arterial pressure, and central venous pressure did not change after infusion of 200 ml of 20% albumin. Furthermore, no difference was detected between responders and non-responders [28]. Nearly 60% of patients in the present study were identified responders by increased CO with relatively high CO during PLRT. That was to say, the cause of shock in this population remained partly due to insufficient volume indicating that more fluids should be given. Such facts implied that, firstly, if the patient had received sufficient fluid management; secondly, the following goals of fluid treatment should be made on account of the results after BIT.

PLRT accurately predicts fluid responsiveness [11], yet it has contraindications and disadvantages [2, 29]. The present study aimed to discover a reliable alternative to the PLRT as a complement to several approaches for predicting fluid responsiveness. The blood infusion is a routine at the end of RRT, which does not require an additional operation. Besides, echocardiography is also a noninvasive, point-of-care measure. The present study sought to verify the predictive value of VTI for hemodynamic parameters, such as CO by PiCCO. Moreover, we anticipated the complementary role of BIT for the conventional PLRT to predict fluid responsiveness. The mechanism was that increased preload boosted stroke volume and therefore reflect the fluid responsiveness. According to our observation, CO returned to a similar baseline level after PLRT and before BIT started. It could be potentially explained by the comparable volume infusion. Volume from both lower extremities was the only factor that could have an influence on CO when the vasoactive agents and cardiac contractile function were kept at the same level.

Fluid responsiveness is the basis of fluid resuscitation, while responders do not necessarily imply fluid expansion. When the patient's circulation is relatively stable, i.e., stabilization and de-escalation [7], fluid management is preferable to merely volume expansion. In the present study, patients were elderly with an average age of 87 years old, taking into account the risk of pulmonary edema, ascites, or multiple organ dysfunction syndrome, we decided on a rather safe evaluation for fluid responsiveness at the end of RRT. It was a common clinical scenario that fluid responsiveness was evaluated on the premise of maintaining organ perfusion rather than before or during resuscitation. Fluid responsiveness can not only be measured prior to fluid expansion but also benefit critically ill patients undergoing RRT for continuous monitoring and balancing fluid in the body, including intravenous medication and nutrition.

### Limitations

The present study employed PLRT as ‘the golden standard’ instead of the fluid challenge. Still, we insisted that sometimes fluid challenge was not necessary and might be hazardous to patients with unstable hemodynamics.

## Conclusions

BIT served as a prediction test at the end of RRT which might guide the individualized fluid management in the following therapeutic schedule.

## Supplementary Information

**Additional file 1.**

## Data Availability

All data generated or analyzed during this study are included in this published article.

## Abbreviations

RRT: Renal replacement therapy
BIT: Blood infusion test
PLRT: Passive leg raise test
VTI: Velocity time integral
PiCCO: Pulse Contour Cardiac Output
CO: Cardiac output
ICU: Intensive care unit
CVVHDF: Continuous venovenous hemodiafiltration
CVVH: Continuous venovenous hemofiltration
LVOT: Left ventricular outflow tract
ROC: Receiver-operating characteristic
