# Supplementary material for: Changes of cardiac output and velocity time integral in blood return at the end of renal replacement therapy predict fluid responsiveness in critically Ill patients with acute circulatory failure
Source: BMC Anesthesiol. 2023 Jan 14;23:25. doi: 10.1186/s12871-023-01976-7 (PMC9840273; doi:10.1186/s12871-023-01976-7)
Supplement: Supplementary file 1 — Additional file 1. [file 12871_2023_1976_MOESM1_ESM.docx]

Table S1.1 contingency table evaluating the accuracy of velocity time integral (VTI) during blood infusion test (BIT) for determining fluid responsiveness.

|  | PiCCO during PLRT | |  |
| --- | --- | --- | --- |
| Echocardiography during BIT | △CO≥10% | △CO＜10% | Total |
| △VTI≥9% | 23 | 2 | 25 |
| △VTI＜9% | 2 | 16 | 18 |
| Total | 25 | 18 | 43 |

PiCCO, Pulse index Contour Cardiac Output; CO, cardiac output; PLRT, passive leg raise test. The diagnostic accuracy was measured using sensitivity (92%), specificity (88.9%), positive predictive value (92%), negative predictive value (88.9%), positive likelihood ratio (8.29), negative likelihood ratio (0.09), accuracy (90.7%), and Youden's index (0.81).

Table S1.2 contingency table evaluating the accuracy of cardiac output (CO) during blood infusion test (BIT) for determining fluid responsiveness.

|  | PiCCO during PLRT | |  |
| --- | --- | --- | --- |
| PiCCO during BIT | △CO≥10% | △CO＜10% | Total |
| △CO≥10% | 14 | 1 | 15 |
| △CO＜10% | 1 | 14 | 15 |
| Total | 15 | 15 | 30 |

PiCCO, Pulse index Contour Cardiac Output; PLRT, passive leg raise test. The diagnostic accuracy was measured using sensitivity (93.3%), specificity (93.3%), positive predictive value (93.3%), negative predictive value (93.3%), positive likelihood ratio (13.93), negative likelihood ratio (0.07), accuracy (93.3%), and Youden's index (0.87).
